# Novel photonic methods for diagnosis of SARS‐CoV‐2 infection

**DOI:** 10.1002/tbio.202200001

**Published:** 2022-03-15

**Authors:** Naveen Joshi, Shubhangi Shukla, Roger J. Narayan

**Affiliations:** ^1^ Department of Materials Science and Engineering North Carolina State University Raleigh North Carolina USA; ^2^ Joint Department of Biomedical Engineering North Carolina State University Raleigh North Carolina USA

**Keywords:** advanced microscopy, aggregation‐induced emission, covid‐19 diagnosis, molecular spectroscopy, nanomaterials, photonic methods, surface enhanced infrared spectroscopy

## Abstract

The COVID‐19 pandemic that began in March 2020 continues in many countries. The ongoing pandemic makes early diagnosis a crucial part of efforts to prevent the spread of SARS‐CoV‐2 infections. As such, the development of a rapid, reliable, and low‐cost technique with increased sensitivity for detection of SARS‐CoV‐2 is an important priority of the scientific community. At present, nucleic acid‐based techniques are primarily used as the reference approach for the detection of SARS‐CoV‐2 infection. However, in several cases, false positive results have been observed with these techniques. Due to the drawbacks associated with existing techniques, the development of new techniques for the diagnosis of COVID‐19 is an important research activity. We provide an overview of novel diagnostic methods for SARS‐CoV‐2 diagnosis that integrate photonic technology with artificial intelligence. Recent developments in emerging diagnostic techniques based on the principles of advanced molecular spectroscopy and microscopy are considered.
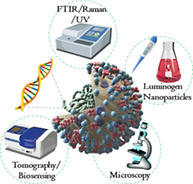

## Graphical abstract

The development of a reliable, rapid, and low‐cost approach with increased sensitivity for the detection of SARS‐CoV‐2 virus and COVID‐19 disease is an important priority of the scientific community. This review provides an overview of photonic approaches that are being explored for the detection of SARS‐CoV‐2 infection and COVID‐19 disease.

## 1 INTRODUCTION

**FIGURE 1:**
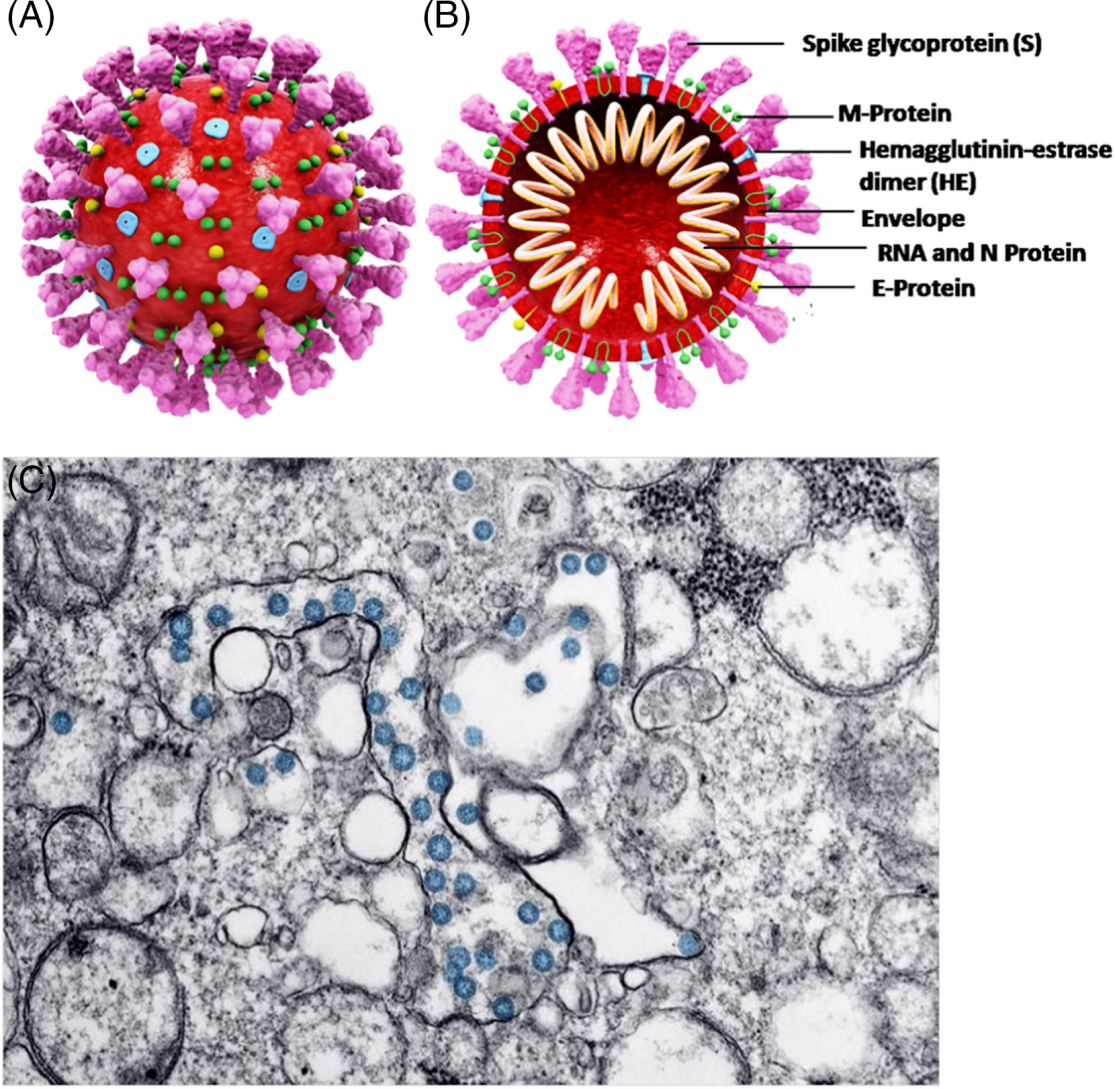
(A) 3D Structure of SARS‐CoV‐2. (B) cross‐sectional representation of the viral structure with its proteins. (C) Transmission electron microscopy image of SARS‐CoV‐2 virus. The virus is colorized in blue. Reprinted from Ref. [], Copyright (2021), with permission from Elsevier

Coronaviruses are large, single‐stranded, enveloped RNA viruses that are responsible for causing respiratory tract infections in humans [1]. Samples collected from a group of unidentified pneumonia patients from Wuhan, China, in December 2019 revealed the presence of a novel coronavirus, which the International Committee of Taxonomy of Viruses termed Severe Acute Respiratory Syndrome Coronavirus‐2 (SARS‐CoV‐2); the corresponding disease is referred to as Corona Virus Disease‐2019 (COVID‐19) [2]. The SARS‐CoV‐2 pathogenic strain is a combination of Middle East Respiratory Syndrome coronavirus (MERS‐CoV) and SARS‐CoV viruses. SARS‐CoV‐2 is known to infect humans via the angiotensin‐converting enzyme 2 (ACE2) receptor; the virus contains a spike protein that binds to the host cell through ACE2, which is located on the cell surface of the host membrane as shown in Figure 1. The clinical and pathological symptoms of COVID‐19 resemble SARS, which is caused by SARS‐CoV [3]. Several variants of concern have emerged, including those identified as alpha, beta, gamma, delta, and omicron by the World Health Organization, since November 2020. These SARS‐CoV‐2 variations are associated with mutations to the viral genome. One recent variant, which was identified as omicron by the World Health Organization, is associated with greater immune escape activity from protection associated with vaccination or prior infection as well as an increased transmission rate due to increased affinity to the ACE2 receptor. The common symptoms noted among COVID‐19 patients include high fever, severe headache, dry cough, and shortness of breath. Individuals with SARS‐CoV‐2 infection exhibit a variety of symptoms from mild respiratory tract infection to chronic respiratory disease; critically ill cases may be associated with acute respiratory distress syndrome and death [4].

The detection of the presence of the SARS‐CoV‐2 virus is a crucial step in responding to the COVID‐19 pandemic as it plays a vital role in isolating infected individuals and controlling viral transmission. Up to the present time, diagnostic methods developed for the detection of the COVID virus have been based on the nucleic acid polymerase chain reaction (PCR) coupled with serological tests such as the detection of immunoglobin M (IgM) and immunoglobin G (IgG) [6]. Oropharyngeal and nasopharyngeal swab samples are often used to collect patient specimens; enzyme linked immunosorbent assay (ELISA) and reverse transcriptase‐polymerase chain reaction (RT‐PCR) are widely used in clinics and hospitals around the world to quantify IgM and IgG levels in these specimens. A comprehensive list of several currently available diagnostic methods is shown in Table 1.

**TABLE 1:** Various diagnostic methods currently in use for the detection of SARS‐CoV‐2

Test type	Limit of detection	Sensitivity (%)	Specificity (%)
Real time RT‐PCR	250‐500 copies/mL	100	100
Multiplex real time RT‐PCR	330‐500 copies/mL	100	100
Isothermal amplification (nucleic acid)	4 viral copies/μL	100	99
Lateral flow immunoassay (LIFA)	—	96	99.6
Luminescent assay	—	100	99.8
ELISA	5 IU/mL	100	100

*Note*: Reprinted in a modified form Ref. [13], Copyright (2021), with permission from Elsevier.

However, these methods are expensive, time consuming, and not accurate at all times. Due to the shortage of ELISA and RT‐PCR testing facilities, samples must be transported to the available centers, which further increases the cost and time delay associated with testing. Photonic diagnosis techniques may offer an alternative to conventional techniques for rapidly diagnosing SARS‐CoV‐2 infection without the need for skilled human labor; in addition, these techniques are associated with enhanced sensitivity, low cost, and low power consumption [7, 8, 9]. Photonic techniques involve the use of light for energy generation, detection, and/or the transmission of information; microscopy and optical communications techniques are common examples of photonic techniques. Recent studies have explored the development of biophotonic sensors based on spectroscopic emission and the absorption of laser energy [10]. Molecular spectroscopic techniques have enabled researchers to better understand the host response and the diagnosis of viral diseases [11]. Functionalized nanoparticles embedded with spectroscopic tools provide valuable information about the presence of viruses with ultra‐high sensitivity. State‐of‐the‐art microscopy techniques can offer direct evidence of the presence of viruses. They can provide direct evidence of interactions between viruses and host cells that cannot be detected via conventional diagnostic tools. When a novel virus needs to be detected, specific probes may not be available to generate molecular and serological assays for detection of the virus; on the other hand, direct visualization via microscopic techniques may allow for rapid identification of the virus [12]. This review focuses on some of the emerging spectroscopic and microscopic techniques for detecting the SARS‐CoV‐2 virus and diagnosing COVID‐19 disease.

## 2 MOLECULAR SPECTROSCOPIC TECHNIQUES

Molecular spectroscopy techniques involve the interaction between electromagnetic radiation and biological entities. Body fluids such as saliva, blood, and urine can be studied with great precision using vibrational and electronic spectroscopic techniques such as infrared spectroscopy and optical biosensing. For instance, protein structures can be studied by analyzing the vibrational bands associated with the protein structure. Vibrational spectroscopy and imaging provide valuable information regarding molecular level interactions without the need for stains or dyes. Furthermore, these techniques require small sample sizes and can be performed accurately without skilled human labor [9, 14, 15]. This section reviews some of the hybrid spectroscopic techniques that are being explored for the detection of SARS‐CoV‐2 virus.

### 2.1 Surface‐enhanced infrared absorption spectroscopy

Surface‐enhanced infrared absorption spectroscopy (SEIRAS) is a surface sensitive technique in which biological molecules are identified by exploiting the electromagnetic attributes of nanostructured metal films, which enhance the vibrational fingerprints of the molecules [16]. Initially, the molecules are adsorbed on the conducting (metal) surface of the thin film. When the infrared rays are irradiated on the adsorbed molecules, the vibrational modes of molecules induce the dipolar modes in the nanostructured metal particles. These molecular vibrations can be coupled with the neighboring nanoparticles in the near field, enhancing infrared absorption [17]. As such, small amounts of analyte molecules can be detected with high sensitivity through an increase in infrared absorption. Owing to its speed and accuracy, SEIRAS is widely used to study surface photochemistry and catalytic reactions as well as to detect various biological molecules [18, 19]. For example, Yao et al. demonstrated the use of the SEIRAS effect to detect SARS‐CoV‐2 by analyzing the interaction of SARS‐CoV‐2‐containing samples with single‐stranded DNA probes that were functionalized on the surface of evaporated gold nano‐island films. The single‐stranded DNA probes contained genomic sequences associated with SARS‐CoV‐2. The SEIRAS spectra from viral RNA‐containing samples were compared with those from control samples without viral RNA; this approach enabled the detection of 1 μM of SARS‐CoV‐2 nucleic acid without amplification in under 5 minutes [16]. In addition, SEIRAS combined with recombinase polymerase amplification treatment enabled detection of 5 am of SARS‐CoV‐2 nucleic acid in 30 minutes.

### 2.2 Fourier transform infrared spectroscopy

**FIGURE 2:**
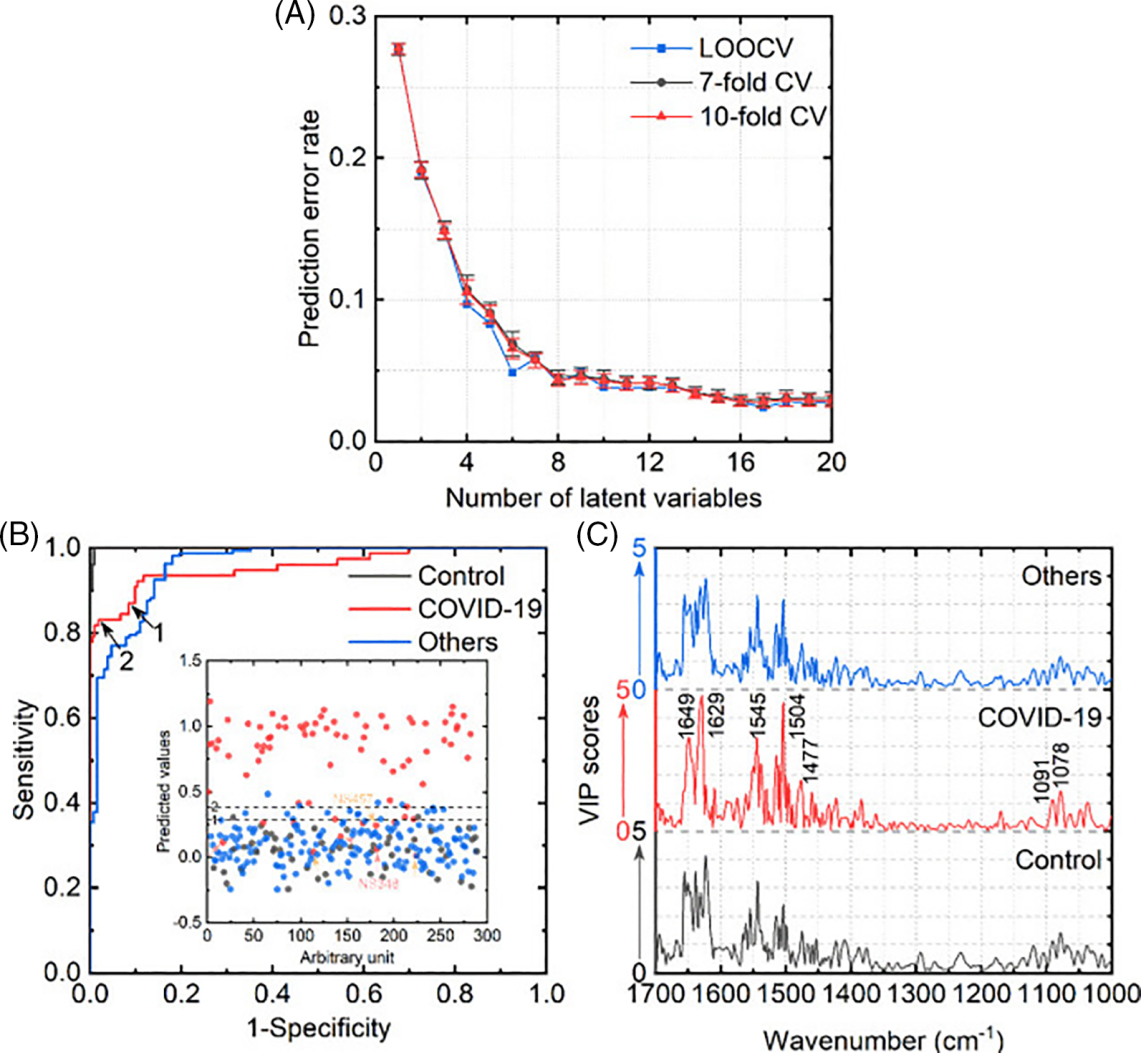
PLS‐DA model performances for the triple‐class classification. (A) Prediction error rates as a function of the number of latent variables. For sevenfold and 10‐fold cross‐validation, the error bars were presented. (B) ROC graphs for each group. The inner graph shows the model predicted output of the COVID‐19 class. The decision threshold values of 1 and 2 are 0.288 and 0.383, respectively. (C) VIP scores for each class. Significant peaks were labeled. Reprinted from Ref. [21], Copyright (2021), with permission from ACS Publications

The use of infrared spectroscopy for COVID‐19 disease diagnosis is relatively new. Attempts have been made to couple spectroscopic tools with artificial intelligence models in order to detect the SARS‐CoV‐2 virus. Kitane et al. developed a technique to detect SARS‐CoV‐2 using extracted RNA samples by combining FTIR and machine learning (ML) techniques. Nasopharyngeal samples collected from 280 patients were processed to extract the RNA. The FTIR spectral domains lying at 600‐1350 cm^−1^; 1500‐1700 cm^−1^; and 2300‐3900 cm^−1^ were attributed to RNA fingerprints [20]. The derivatives of the raw spectra obtained were used to normalize the data of the transformed spectra; machine learning algorithms were used to build classification models. A large number of samples and sparse classification techniques were used to improve the specificity, sensitivity, and accuracy of the data analysis as well as to enhance the interpretability of models. This approach was noted to take only minutes of testing time after RNA extraction while detecting the SARS‐CoV‐2 virus with 97% sensitivity, 97.8% accuracy, and 98.3% specificity. In another study, Zhang et al employed ATR‐FTIR detection technique to detect COVID‐19 disease in ∼3‐μL serum samples. A combined spectroscopic and statistical analysis was done for COVID‐19 positive samples and control samples. The partial least‐squares‐discriminant analysis (PLS‐DA) helped to differentiate SARS CoV‐2 viral strain from inflammation or other respiratory viral infections. This approach provided an area under the receiver operating characteristic curve (AUROC) value of 0.956 [21]. Similarly, Banerjee et al applied the ATR‐FTIR acquisition method in sequence with partial least‐squares‐discriminant analysis (PLS‐DA) models. Consideration of the ATR‐FTIR spectra and the clinical parameters (eg, sex, age, hypertension status, and diabetes mellitus status) increased the area under the ROC curve (AUC), which indicates how well the parameter can distinguish between diseased and normal, for both the training data and test data. The independent test set achieved 94.1% sensitivity and 69.2% specificity. Samples from diabetes mellitus patients, FTIR region 1020‐1090 cm^−1^, and FTIR region 1588‐1592 cm^−1^ were the strongest predictors [22]. Guleken et al studied the detection of COVID‐19 disease in the blood serum of symptomatic and asymptomatic and pregnant women. The samples from pregnant women with COVID‐19 disease and healthy pregnant women were compared [23]. The samples were analyzed using FTIR; the peak shifts were analyzed with multivariate machine learning approaches (eg, a Random Forest algorithm, a C5.0 single decision tree algorithm, and a deep neural network). The biochemical levels, peripheral blood cell levels, and coagulation parameters for pregnant women are shown in Figure 2. The machine learning techniques were able to differentiate among the groups using amide II vibrations, amide I vibrations, and CH_2_ scissoring; an accuracy greater than 90% was demonstrated using this approach [23].

### 2.3 Emission spectroscopy

The qualitative and quantitative sensing of antigens and antibodies associated with SARS‐CoV‐2 infection can rely on immunological assays; these assays exploit the luminescence properties of various probes [24]. Novel probes with enhanced sensitivity can improve the performance of these assays. Aggregation‐induced emission (AIE) luminogens offer enhanced sensitivity for the detection of molecules in the aggregated states. Hence, they have been considered for the detection of biomolecules and therapeutics. Aggregation‐induced emission was first proposed by Luo et al; this approach is associated with low luminescence in the molecular state and enhanced emission in the aggregated state [25].

This property is utilized to understand viral density, which reaches a maximum value after a few days of infection; understanding the viral density can facilitate early detection of SARS‐CoV‐2 infection. In many cases, commercial double‐stranded DNA dyes show low luminescence in free state. Their luminescence intensity increases alongside the amplification of complimentary DNA (cDNA). This approach reduces the signal‐to‐noise ratio significantly, enhancing the detection sensitivity [26]. AIE luminogens have also been reported to show great potential for use in immunological assays such as enzyme‐linked immunosorbent assay (ELISA) and lateral‐flow immunoassays. ELISA commonly utilizes SARS‐CoV‐2 antibodies to bind the SARS‐CoV‐2 viral proteins and form antibody‐protein complexes, which can be observed using an additional tracer antibody. Guo et al demonstrated the use of an immune capture probe (Fe_3_O_4_@Ab1IgG) containing magnetic Fe_3_O_4_ nanospheres and mouse antihuman IgG (Ab1IgG); a fluorescence detection probe (QBs@Ab2IgG) containing highly luminescent quantum dot nanobeads (QBs) and rabbit antihuman IgG (Ab2IgG) was used for detection with high sensitivity [27]. After a magnetic separation process, the fluorescence intensity of the precipitate was measured using a 370 nm excitation wavelength. A lower limit of detection of 4 pg mL^−1^ was demonstrated using this approach; in addition, the approach was successfully used to study human IgG in serum samples, with results that were not statistically different from commercial enzyme‐linked immunosorbent assays.

### 2.4 Luminogen nanoparticles

**FIGURE 3:**
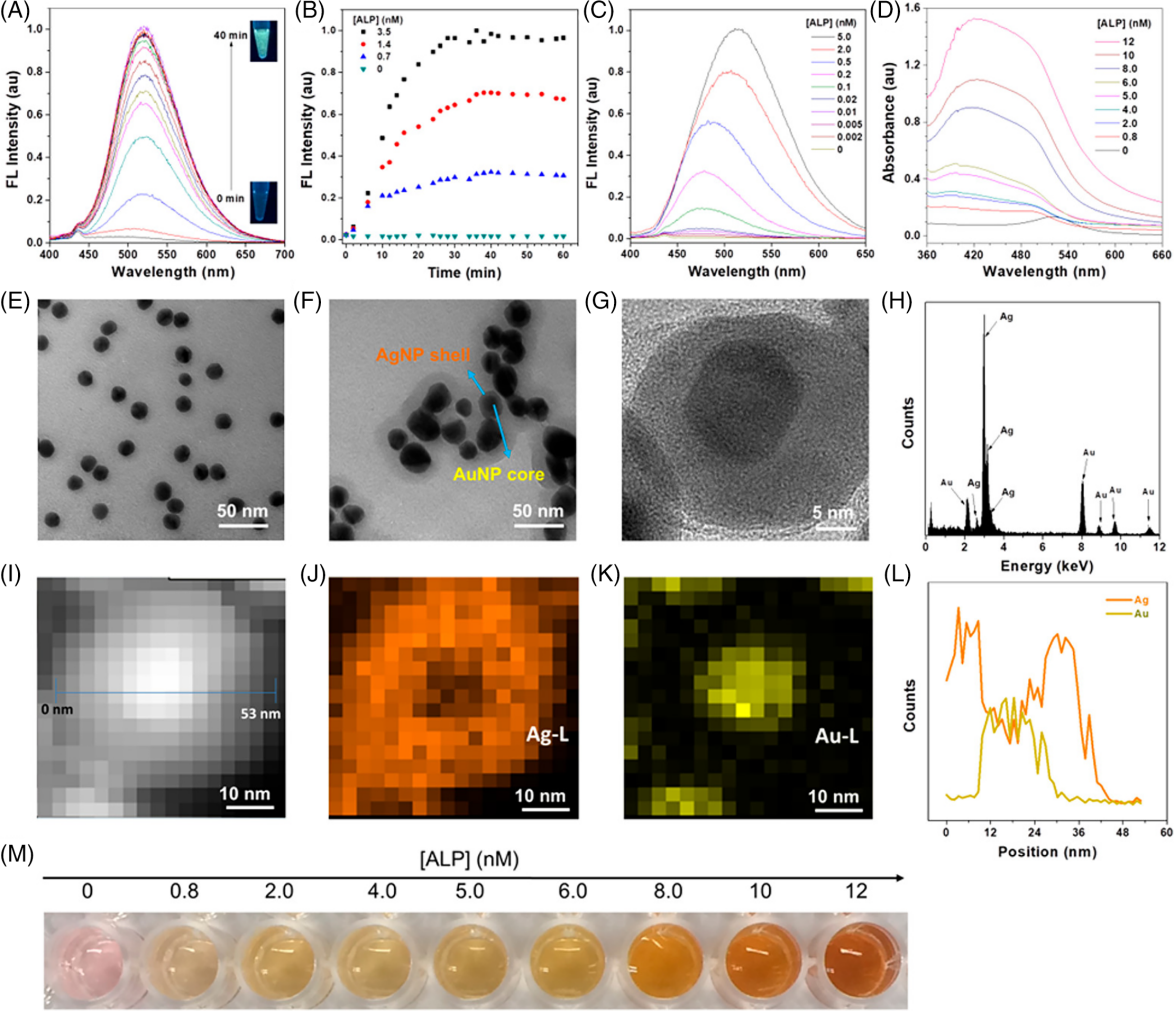
Establishment of fluorescence and plasmonic colorimetry based dual‐modality detection method. (A) Fluorescence spectra evolution of the mixture of TPE‐APP (100 μM) and ALP (3.5 nM) during 40‐minute incubation time. (B) Plotting of fluorescence intensity vs incubation time in the presence of various concentrations of ALP. (C) Fluorescence spectra of the mixture of TPE‐APP (100 μM) and various concentrations of ALP after 30 minutes incubation. (D) Absorption spectra of the mixture of AuNP (3.2 nM), Ag + (1.2 mM), and TPEAPP (100 μM) with various concentrations of ALP after 30 minutes incubation. (E) TEM image of AuNP. (F) TEM image of AuNP@AgNP produced by ALP (6 nM). (G) HRTEM image and (H) EDX profile of AuNP@AgNP. (I) STEM‐HAADF image, (J) Ag element, and (K) Au element mapping of AuNP@AgNP, respectively. (L) EDXS line profile for image (H). (M) Photograph of the color change in (D). Reprinted from Ref. [30], Copyright (2018), with permission from ACS Publications

Aggregation‐induced emission‐luminogen‐based nanoparticles or aggregation‐induced emission dots are being explored as potential candidates for the detection of biomolecules with good luminescence, water solubility, photostability, and biocompatibility properties [28]. They are prepared by precipitating aggregation‐induced emission luminogens in an anti‐solvent. Sophisticated molecular designs are employed to control the particle morphology and size to improve the functionality of the nanoparticles. Lateral‐flow immunoassays are commonly used for SARS‐CoV‐2 detection; in this approach, a paper‐like membrane containing two strips is used to conduct the test. The test strip of the membrane is coated with fluorescent material‐antibody conjugate, and the control strip of the membrane is coated with capture antibodies. When a blood sample is placed on the membrane strip, SARS‐CoV‐2 antibodies attach to the fluorescent material‐antibody conjugates within the test strip. These complexes are subsequently immobilized by the capture antibodies on the control strip to generate a visible line. Zhang et al demonstrated immunochromatographic assays that contained aggregation‐induced emission fluorescent microspheres. By integrating AIE luminogens into fluorescent microspheres, much higher relative quantum yield and maximum fluorescence intensity yield values than those from conventional immunochromatographic assays from Ocean or Merck for the detection of Escherichia coli O157:H7 were obtained [29]. In another study, a dual‐modality readout immunoassay platform based on multifunctional aggregation‐induced emission luminogens was demonstrated for ultrasensitive virus detection; this approach involved virion‐based immuno‐bridged enzymatic hydrolysis of the multifunctional aggregation‐induced emission luminogens. In particular, the formation of aggregates and shelling of silver on gold nanoparticles was observed. It was noticed that the reduction of silver ions leading to the formation of silver nanoshells on the surface of gold nanoparticles led to turn‐on fluorescence and a plasmonic color change (as shown in Figure 3) with ultrasensitive detection capabilities [30]. EV71 virions were detected within 24 clinical samples with 100% accuracy using this approach. Tian and coworkers envisage new developments in the clinical diagnosis of SARS‐CoV‐2 infection with high sensitivity by varying aggregation‐induced emission luminogens and tuning the luminescence of aggregation‐induced emission dot composites [31].

### 2.5 Single‐photon emission computed tomography

With the advancements in molecular and radiological diagnostics, single‐photon emission computed tomography (SPECT) is considered one of the powerful imaging techniques to examine the blood flow to tissues and organs. It is a nuclear imaging technique that combines computed tomography and the use of radioactive tracers. The latter helps in detecting the blood flow to the organs to diagnose infections, tumors, seizures, and other medical conditions [32]. Typically, fluorine‐18, iodine‐123, thallium‐201, and xenon‐133 are the radioisotopes used in this technique. Das et al showed that perfusion (Q)‐single‐photon emission computed tomography was useful in diagnosing pulmonary embolus in a group of six patients with SARS‐CoV‐2 infection [31]. Vöö and Dizdarevic indicated that the integration of SPECT with low‐dose computed tomography is helpful for diagnosing venous thromboembolic events in COVID‐19 patients; SPECT perfusion only may be used in pregnant COVID‐19 patients [33]. Cobes et al indicated that SPECT with low‐dose computed tomography may be used for the diagnosis of pulmonary embolism in patients being treated for COVID‐19 pneumonia through the observation of anomalies in ventilatory and perfusion [34].

### 2.6 Optical biosensors

Optical biosensors are widely explored as point of care (POC) diagnostics for viral sensing because of their relative safety and low cost of manufacturing. In addition, the use of POC diagnostics does not require trained personnel [35]. As noted by Saylan et al, direct optical biosensors contain a transducer surface in which a signal is generated by the formation of a complex on the surface of the transducer. In an indirect optical biosensor, a chromophore, fluorophore, or other label is used to identify a binding event and amplify the signal that is associated with the event; indirect optical biosensor limitations include high reagent cost and signal interference from nonspecific binding [9]. The recent focus has been on detecting the virus particles by sandwiching them between a peptide sequence and an antibody; Chen et al and Wu et al indicated the occurrence of antibodies that bind to SARS‐CoV‐2. Another target is the spike protein in SARS‐CoV‐2 that interacts with host cells [36, 37]. Maddali et al have suggested the development of optical biosensors SARS‐CoV‐2 receptors for diagnosing COVID‐19 disease [38].

## 3 MICROSCOPIC TECHNIQUES

Microscopy is a powerful tool because of its potential to quantitatively assess the complex dynamics of biological systems. Furthermore, microscopy has played a vital role in infectious disease research since microscopic imaging of microorganisms was demonstrated [39]. It also had a major role in identifying pathogens and in the diagnosis of infectious diseases. For example, Electron microscopy (EM) is used for the direct visualization of viruses. In fact, it remains the only technique being able to provide comprehensive information on the structure and morphology of viral proteins on the virion surface [40]. As such, EM has been utilized for understanding the pathogenesis of the SARS‐CoV‐2 virus. The earliest reports on the identification of SARS‐CoV‐2 strongly relied on microscopy data to provide evidence for the emergence of the virus. Digitally enhanced electron microscopic images of the SARS‐CoV‐2 virions have been widely used by the news media as resources to raise awareness of the COVID‐19 pandemic [41].

### 3.1 Photonic resonator absorption microscopy

**FIGURE 4:**
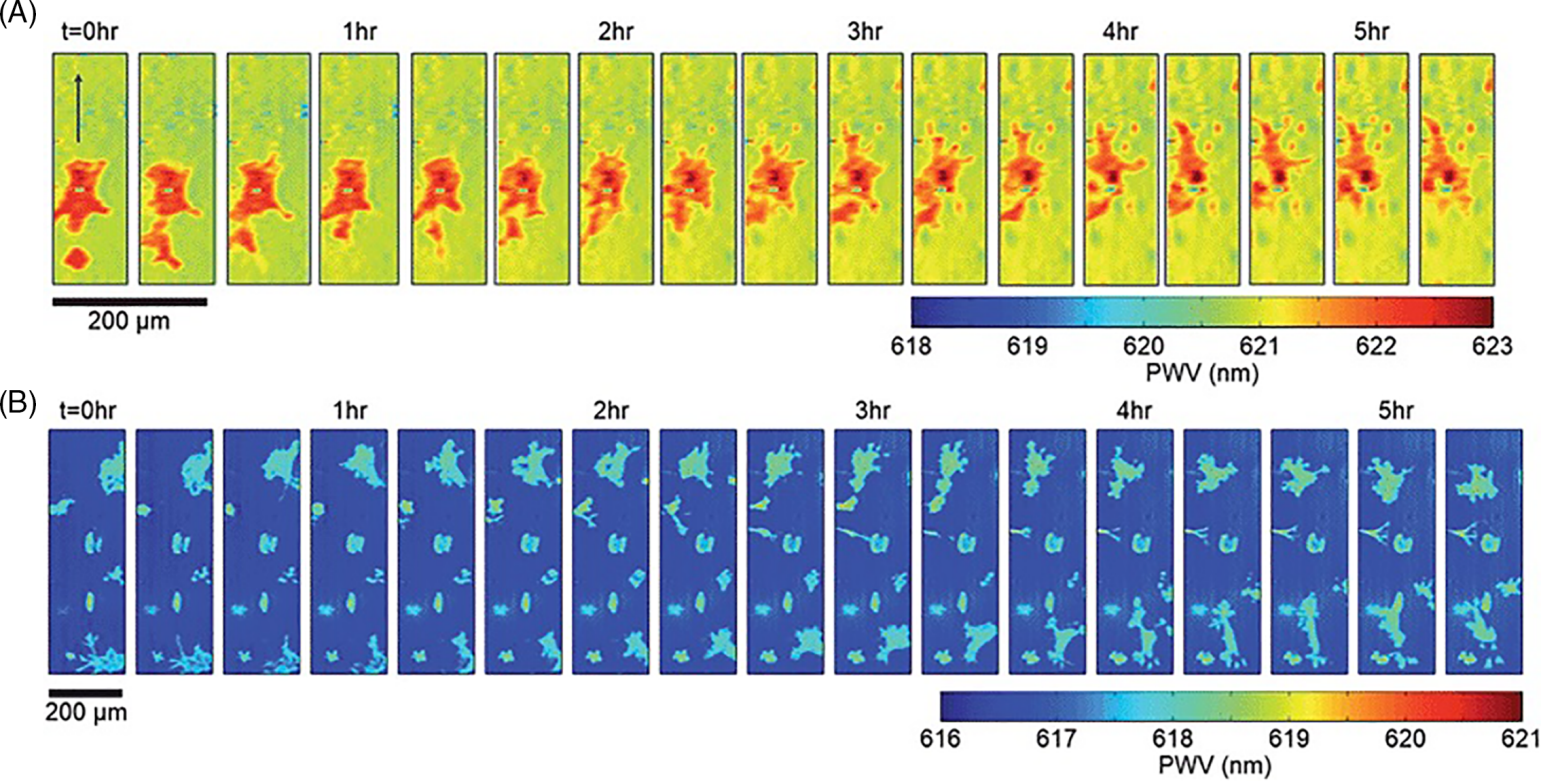
Time lapse PWV images of chemotaxis of mHAT9a cells. Cells were deposited on the sensor surface at a concentration of 8000 cells per mL and allowed to attach for 2 hours before imaging. An agarose bead was placed at a location approximately 100 μm above the top of the image, and PWV images were collected every 20 minutes after the bead was placed. Cell movement direction is indicated with an arrow in the leftmost frame. (b) CXCR4 knockout cells exhibit non‐directional movement on the sensor surface. Similarly prepared, CXCR4 mutants do not show directional movement toward the bead, demonstrating that the previously observed directional locomotion was due to chemotaxis. Reprinted from Ref. [47], Copyright (2013), with permission from RSC Publications

Photonic crystals are optical resonators that are being widely explored for digital resolution microscopy and biosensing [42, 43]. Photonic crystals contain a periodic arrangement exhibiting dielectric permittivity that acts as an optical analog for the atomic potential for electrons [44]. Resonant wavelengths are generated by manipulating the electromagnetic fields associated with the light emitted from photonic crystals. As such, an electromagnetic standing wave showing intensities relative to the intensities of an illuminating light source is generated [45, 46, 47]. Furthermore, it has been demonstrated that gold nanoparticles (AuNP) with localized surface plasmon resonance (LSPR) overlap with the resonant electromagnetic field of a photonic crystal, resulting in a significant amplification of the absorption efficiency [48]. This combination of gold nanoparticles and photonic crystals surface enables the observation of individual gold nanoparticles with a conventional inverted optical microscope; in this approach, enhanced nanoparticle absorption attenuates light that is reflected into the microscope objective. Photonic resonator absorption microscopy is a type of biosensor microscopy that utilizes photonic crystals‐AuNP resonant coupling [49, 50, 51]. As noted by Canady et al, the reflected light intensity from the photonic crystals is locally quenched in the presence of each nanoparticle when the surface plasmon‐resonant wavelength of the nanoparticle matches the resonant wavelength of the photonic crystal [49]. They demonstrated ultrasensitivity (<1 pm) as well as single‐base mismatch selectivity and 100‐aM limit of detection via a kinetic discrimination assay [49]. Chen et al studied the use of a label‐free, submicron resolution approach called photonic crystal enhanced microscopy, which utilizes photonic crystal biosensor surface as a substrate for cell attachment, to understand the attachment and morphology of murine dental stem cells. The time lapse study involving chemotaxis with a chemoattractant is shown in Figure 4 [46]. Zhao et al studied the use of a photonic crystal optical biosensor coated with a recombinant spike protein for the quantitative detection of serological human IgG levels against the SARS‐CoV‐2 virus. This single‐step, wash‐free approach uses antibody‐functionalized gold nanoparticles that form sandwich immunocomplexes; an approach termed as “Active Control + Digital Counting” (AC + DC) enabled the detection of 100 pg mL^−1^ of human COVID‐19 IgG in serum samples during a 15‐minute assay [50]. Ghosh et al described the use of photonic resonator absorption microscopy with illumination from a polarized light emanating from a low‐intensity red LED and detection with an inexpensive CMOS image sensor for inexpensive point of care biosensing; they demonstrated the detection of miRNA sequences with a 160 am detection limit with a 30‐minute assay time [52].

### 3.2 High‐throughput microscopy in antibody diagnostics

High‐throughput microscopy utilizes cell‐based assays along with fluorescently labeled molecular components and quantification via image analysis methods. This approach is used to understand host‐pathogen interactions and evaluate antimicrobial compounds [53]. In addition, it is widely used in genome‐wide genetic screening using RNA interference. One benefit of high‐throughput microscopy over conventional biochemical techniques is that mechanisms associated with cell penetration and intracellular metabolism are more predictive using cellular assays than using biochemical assays. Furthermore, it is possible to assess toxicity and modes of action using a single assay instead of using multiple assays [54]. For example, high‐throughput microscopy may be used as a cellular assay to monitor SARS‐CoV‐2 virus infectivity. Froggatt et al. developed an approach for rapid drug discovery based on a green fluorescent protein (GFP)‐derived protein that changes fluorescent properties upon cleavage by the viral protease, 3CLpro, which is a target of antiviral therapies. Changes in fluorescence reflect the activity of protease and can be used to identify inhibitors of the SARS‐CoV‐2 protease with high throughput [55].

### 3.3 Electron microscopy

EM accompanied by immunolabeling is being utilized to evaluate viral levels in body fluids and excretions [56]. For example, plasma and stool samples were used to identify hepatitis B9 and A10; BK virus, a type of papovavirus, was first observed on cells in the ureter using EM [57, 58]. However, cellular structures such as ribosome‐decorated endoplasmic reticulum membranes and intraluminal vesicles can be misidentified as SARS‐CoV‐2 viral particles [59, 60, 61]. Autolysis of structures in autopsy samples can present challenges during EM‐based identification of virus‐associated cellular changes [60]. Cortese and Laketa proposed that transmission EM together with immunolabeling is useful as a diagnostic approach for detecting viruses in body fluids and feces [12]. SARS‐CoV‐2‐infected human lung epithelial cells showed organelle alterations via EM; in particular, disintegration of the Golgi apparatus, modification of the mitochondria, and formation of viral replication organelles containing double‐membrane vesicles were noted [62].

## 4 CONCLUSIONS

Due to their versatility, photonic approaches are useful for diagnosing nascent outbreaks. The development of rapid and accurate photonic techniques may also assist with mitigating the spread of ongoing outbreaks. This review provides an overview of emerging photonic methods that are being explored for the detection of SARS‐CoV‐2 infection and COVID‐19 disease. Novel diagnostic methods based on molecular spectroscopy and microscopy for the detection of the SARS‐CoV‐2 infection are being considered. Future efforts that enhance specificity, sensitivity, and speed are needed to assist with the clinical translation of photonic diagnosis approaches.

## Data Availability

Research data are not shared.
